# Odontogenic Differentiation-Induced Tooth Regeneration by *Psoralea corylifolia* L.

**DOI:** 10.3390/cimb44050156

**Published:** 2022-05-19

**Authors:** Hye-Ock Jang, Tea-Young Ahn, Ji-Min Ju, Soo-Kyung Bae, Hyung-Ryong Kim, Da-Sol Kim

**Affiliations:** 1 Department of Dental Pharmacology, School of Dentistry, Pusan National University, Yangsan 50612, Korea; jho9612@pusan.ac.kr (H.-O.J.); kuen001@naver.com (T.-Y.A.); wnwlals88@gmail.com (J.-M.J.); skbae@pusan.ac.kr (S.-K.B.); 2 Dental and Life Science Institute, School of Dentistry, Pusan National University, Yangsan 50612, Korea; 3 Education and Research Team for Life Science on Dentistry, Pusan National University, Yangsan 50612, Korea; 4 Department of Pharmacology, College of Dentistry, Jeonbuk National University, Jeonju 54896, Korea; 5 Department of Dermatology, Pusan National University Yangsan Hospital, Yangsan 50612, Korea

**Keywords:** traditional medicines, *Psoralea corylifolia* L., dental pharmacology, odontoblast, tooth regeneration, cell signaling

## Abstract

*Psoralea corylifolia* L. (*P. corylifolia*) has been used as an oriental phytomedicine to treat coldness of hands and feet in bone marrow injury. Hydroxyapatite is usually used for tooth regeneration. In this study, the role of *P. corylifolia* and bakuchiol, a compound originated from *P. corylifolia* as differentiation-inducing substances for tooth regeneration, was determined by monitoring odontogenic differentiation in human dental pulp stem cells (hDPSCs). We confirmed that *P. corylifolia* extracts and bakuchiol increased the odontogenic differentiation of hDPSCs. In addition, the expression of the odontogenic differentiation marker genes alkaline phosphatase (APL), Runt-related transcription factor 2 (RUNX-2), osteocalcin (OC), and dentin matrix acidic phosphoprotein-1 (DMP-1) was proved by real-time polymerase chain reaction, and protein expression of dentin matrix acidic phosphoprotein-1 (DMP-1) and dentin sialophosphoprotein (DSPP) was proved by western blotting. Further, by confirming the increase in small mothers against decapentaplegia (SMAD) 1/5/8 phosphorylation, the SMAD signaling pathway was found to increase the differentiation of odontoblasts. This study confirmed that *P. corylifolia* L. extracts and bakuchiol alone promote odontogenic differentiation in hDPSCs. These results suggest that bakuchiol from *P. corylifolia* is responsible for odontogenic differentiation, and they encourage future in vivo studies on dentin regeneration.

## 1. Introduction

Regeneration of a functional and natural tooth is an essential therapeutic strategy for the replacement of a diseased or damaged tooth. Since autogenous tooth regeneration and then replacement is safer than the influx of foreign substances, research on the preservation of autologous teeth is more important than that on tooth replacement with foreign substances. Since the advent of the 2017 tooth regeneration study by the PT Sharpe group [1], using glycogen synthase kinase 3 beta (GSK-3b) inhibitor, various studies on inducing self-tooth regeneration have been conducted.

Stem cells have several features, such as self-renewal and differentiating into multiple mature cell types [2,3,4,5,6,7,8], and there is a small population of these cells that exists in every organ [9]. Mesenchymal stem cells (MSCs) have several specific features; they can form a colony from one cell and are multi-potent, but not totipotent. Therefore, MSCs can differentiate into osteocytes, adipocytes, and cartilage tissues. There are many well-known markers for MSCs, including CD73, CD90, CD105, c-kit, CD14, CD11b, CD34, CD45, CD19, CD79, and human leukocyte antigen (HLA)-DR negative [9,10].

Cells with these characteristics are also found in the oral cavity. Dental stem cells are tissue specific; various types of MSCs are observed depending on their position in the oral cavity. For example, dental pulp stem cells (DPSCs) can be derived from the inner tooth pulp of adult molars, and periodontal ligament stem cells can be derived from the periodontal ligament [10,11,12,13,14]. Although there are many kinds of dental stem cells, in this study, we selected human dental pulp stem cells (hDPSCs) owing to their high oral population.

*P. corylifolia* is an important medicinal plant long known for its clinical applications such as tonifying the kidney yang, controlling nocturnal emissions during diuresis, warming the spleen to stop diarrhea, and helping in inspiration to relieve asthma. The seeds of *P. corylifolia* have been used as an ancient Hindu remedy for vitiligo [15]. Chopra et al. reviewed a detailed survey of the literature on its botany, phytochemistry and ethnopharmacology along with special emphasis given on pharmacological activities of plant *P. corylifolea* [16].

Many studies have investigated the relationship between human MSCs from various organs and tissues [8,17,18,19,20]. In particular, Shuyu E et al. suggested that *P. corylifolia* is involved in mitogen-activated protein kinase pathways [21]. According to the research results related to bone regeneration in *P. corylifolia*, psoralen, psoralidin, etc., promote bone regeneration in bone marrow-derived cells and are reported as candidates for the treatment of osteoporosis [22,23]. Earlier, Li et al. investigated the role of *P. corylifolia* in odontogenic differentiation of hDPSCs, and the results showed that *P. corylifolia* increased odontogenic differentiation [24].

Recently, bakuchiol and ameroterpene phenol of plant origin, promising a new agent as a complement, enhance the effectiveness of the currently marketed available anti-acne formulations. In addition, it also possesses an excellent safety profile and proves to be non-irritant, non-sensitized and, therefore, can be used throughout the day [25,26]. Psoralen and bakuchiol ameliorated bone resorption via inhibition of AKT and AP-1 pathways activation in vitro [26]. Lee et al. suggested that bakuchiol enhances myogenic differentiation through p38MAPK and MyoD activation, and can be developed into a potential agent to improve muscular regeneration [27]. In addition, based on Zhang’s review, we selected bakuchiol, a compound isolated from *P. corylifolia*, for further evaluation.

Estrogen replacement therapy is utilized as a major regime for the treatment of osteoporosis at present, but long term use of estrogen may cause uterine hyperplasia and hypertension leading to a high risk of endometrial cancer and breast cancer [28]. Bisphosphonates are used to suppress osteoclastic activity and to treat osteoporosis, but induced osteonecrosis of the jaw is increasing [29]. There is an urgent need for research that can induce bone or tooth regeneration without the side effects described above.

In this study, we investigated the role of *P. corylifolia* and bakuchiol in the regulation of DPSC proliferation and differentiation, and attempted to optimize the odontogenic differentiation of hDPSCs.

## 2. Materials and Methods

### 2.1. Plant Material

The dry seeds of *P. corylifolia* were purchased from KMD medicinal herbs Co. (Ulsan, Korea). Their origin is Yunnan (China), dried with ventilation at ambient temperature, and stored at 4 °C until use.

### 2.2. Herbal Extraction

For the preparation of *P. corylifolia* extracts, 2 L of 99.8% methanol was added to 200 g of the sample and the bottle was shaken once a day for 8 days. After obtaining the crude extract, the solution was filtered using a 185-mm filter paper. The solution was further concentrated under reduced pressure in an aqueous bath. The concentrated solution was lyophilized using a freeze dryer (Labconco, Kansas, MO, USA). The extraction yield of *P. corylifolia* was 14.15%. This powder was stored at −20 °C. *P. corylifolia* extracts were dissolved in dimethyl sulfoxide (DMSO) for in vitro studies.

### 2.3. Chemicals and Reagents

Cell counting kit-8 (CCK-8, CCK-3000) from Dongin Biotech (Seoul, Korea), bakuchiol (CFN-99047) from ChemFaces (Wuhan, China), and ascorbic acid (A-4544), β-glycero-2-phosphate (G-9891) and dexamethasone (D-2915) from Sigma-Aldrich (St. Louis, MO, USA) were purchased. For odontogenic differentiation, dexamethasone, β-glycerol 2-phosphate and ascorbic acid were added to Minimum Essential Medium-α supplemented with 10% fetal bovine serum (FBS, Gibco, Grand Island, NY, USA) to obtain final concentrations of 0.1, 10 and 50 μM, respectively.

### 2.4. Cell Culture

hDPSCs were purchased from Lonza (PT-5025, Basel, Switzerland). The cells were isolated from human tooth pulp, with three different primary cells being obtained from three different individuals. The cells were characterized by flow cytometry using the CD105+, CD166+, CD29+, CD90+, CD73+, CD133-, CD34- and CD45- surface antigens. The cells were maintained in StemMACS Media XF containing 1X anti-anti at 37 °C in a 5% CO_2_ incubator.

### 2.5. Cell Proliferation Assay

The in vitro proliferation of hDPSCs was determined using CCK-8 assay. The hDPSCs were seeded into 48-well culture plates (30048, SPL, Gyeonggi-do, Korea) at a density of 1 × 10^4^ cells/well and cultured in StemMACS MSC expansion Media XF (130-101-375, Miltenyi Biote, Bergisch Gladbach, Germany) containing 1X anti-anti (L0010-020, Biowest, Nuaillé, France), and *P. corylifolia* extracts.

The cells were cultured for 4 days at 37 °C in a 5% CO_2_ incubator. Following this, CCK-8 solution was added and the cells were incubated at 37 °C in a 5% CO_2_ incubator for 2 h. Absorbance was measured using an ELX800 spectrophotometer (BioTek, Winooski, VT, USA) at 450 nm. Each experiment was performed in triplicate.

### 2.6. Odontogenic Differentiation

Odontogenic differentiation was induced by culturing cells for 7–21 days in the odontogenic medium described in Section 2.3. Calcification of the extracellular matrix was estimated using 2% Alizarin Red S (ARS) solution (pH 4.3, A-5533, Sigma-Aldrich) for 15 min. To obtain quantitative data, 200 μL of 10% (*w/v*) cetylpyridinium chloride (CPC, C-0732, Sigma-Aldrich) and 10 mM sodium phosphate solution (pH 7.0) were added to the dishes containing the staining solution. The absorbance of the extracted dye was measured at a wavelength of 570 nm.

### 2.7. Real-Time Polymerase Chain Reaction Analysis

Total RNA was isolated using TRIzol reagent (17061, iNtRON Biotechnology Inc., Seongnam, Korea) according to the manufacturer’s instructions and reverse-transcribed into complementary (cDNA) using a First Strand cDNA Synthesis Kit (K-2041, Bioneer, Daejeon, Korea). The primer sequences used are shown in Table 1. Quantitative reverse transcriptase polymerase chain reaction (PCR) was performed using TOPreal™ qPCR 2X PreMIX (RT-500M, SYBR Green with low ROX, Daejeon, Korea) on an ABI 7500 instrument (Applied Biosystems, Foster City, CA, USA). Data analysis was performed using the ∆∆Ct method, and the experiments were repeated three times.

### 2.8. Statistical Analysis

The results are expressed as the mean ± standard error of the mean (SEM) values for more than three independent experiments. Statistical significance was determined between the treatment groups and the positive and negative controls. The *p*-value was calculated using Student’s *t*-test. Each experiment was repeated at least three times to yield comparable results. Values of * *p* < 0.05, ** *p* < 0.02 and *** *p* < 0.01 were considered significant.

## 3. Results

### 3.1. Effect of P. corylifolia Extracts on Cell Viability and Odontogenic Differentiation in hDPSCs

**Figure 1:**
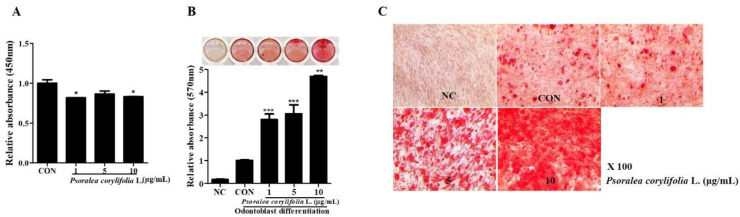
Effect of *P. corylifolia* extracts on proliferation and odontogenic differentiation in hDPSCs (**A**) Cell proliferation was measured at 4 days using the CCK-8 assay. When treated with 1, 5 and 10 μg/mL of *P. corylifolia* extracts, cell proliferation was comparable with the control group. All data are presented as the mean ± SEM. * *p* < 0.05. (**B**) Alizarin red S (ARS) staining was performed on the 10th day of odontogenic differentiation of hDPSCs exposed to *P. corylifolia*. All data are presented as the mean ± SEM (n = 3). ** *p* < 0.02, *** *p* < 0.01. (**C**) Scanned images of plate wells are presented above the quantitation graph of ARS staining. The values obtained for *P. corylifolia* extract-treated group were compared with those of the positive control. (**C**) 100× microscopic images of hDPSCs treated with 1, 5 and 10 μg/mL of *P. corylifolia* extracts. SEM, standard error of mean; NC, negative control; CON, positive control; hDPSCs, human dental pulp stem cells.

To evaluate the effect of *P. corylifolia* extracts on hDPSC odontogenic differentiation, we treated the hDPSCs with *P. corylifolia* extracts during cell proliferation and odontogenic differentiation. We then tested the cellular physiology and the influence on diverse cell types. However, its influence on the differentiation of hDPSCs was inconclusive. The cell proliferation assay showed that cell proliferation was not found to be adversely affected during hDPSC proliferation (Figure 1A).

The results showed that *P. corylifolia* accelerated odontogenic differentiation about three times faster than general differentiation; the transcription of odontoblast-specific genes and proteins was performed to prove the differentiation.

The degree of odontogenic differentiation was measured by ARS staining, and the intensity of staining was quantified using 10% CPC solution (Figure 1B). Figure 1C shows the optical microscopy images at 100× magnification. Thereby, we hypothesized that, during odontoblast differentiation of hDPSCs, *P. corylifolia* extracts upregulate odontogenic differentiation.

**Figure 2:**
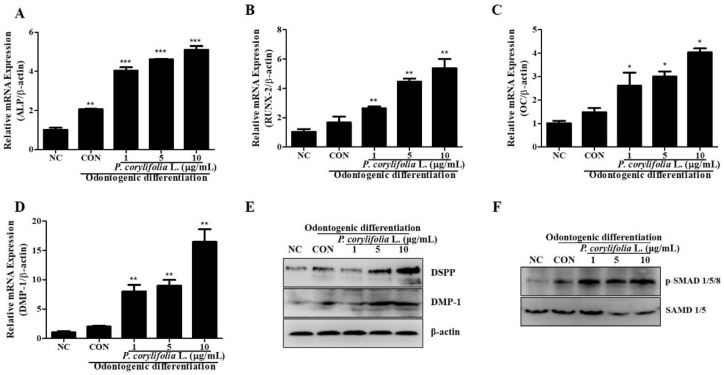
Real-time PCR and western blot analysis during odontogenic differentiation in hDPSCs. (**A**–**D**) Odontogenic differentiation marker gene (APL, RUNX-2, OC, and DMP-1) expression in hDPSCs treated with 1, 5 and 10 μg/mL of *P. corylifolia* extracts. (**E**) Western blot analysis for the expression of odontogenic differentiation protein markers DMP-1 and DSPP expression. (**F**) Western blot analysis to determine the effect of *P. corylifolia* extracts on SMAD 1/5/8 phosphorylation. All data are presented as the mean ± SEM (n = 3). * *p* < 0.05, ** *p* < 0.02, *** *p* < 0.01. SEM, standard error of mean; NC, negative control; CON, positive control; hDPSCs, human dental pulp stem cells; PCR, polymerase chain reaction.

As mentioned previously, in the presence of *P. corylifolia* extracts, hDPSCs display accelerated odontogenic differentiation. To evaluate whether odontogenic differentiation was indeed upregulated under our experimental conditions, the expression of odontogenic differentiation markers was measured using real-time PCR. For real-time PCR, hDPSCs were differentiated and RNA was harvested on day 7 of differentiation after treatment with *P. corylifolia* extracts. cDNA (2 μg) was synthesized from the extracted RNA. ALP, RUNX-2, OC, and DMP-1 were the odontogenic differentiation markers assessed. hDPSCs exposed to *P. corylifolia* extracts for 7 days displayed upregulation of the odontogenic differentiation markers compared to the control (Figure 2A–D). Additionally, Western blot analysis was performed to evaluate DMP-1 and DSPP protein expression; the *P. corylifolia* extract-treated group showed increased DMP-1 and DSPP protein expression (Figure 2E). Several studies have reported that SMAD signaling is a crucial signaling pathway in osteogenic and odontogenic differentiation [30,31,32]. To confirm the cell signaling, SMAD phosphorylation was evaluated. The results showed that *P. corylifolia* extracts increased the odontogenic differentiation of hDPSCs via SMAD signaling (Figure 2F).

### 3.2. Effect of Bakuchiol on Cell Viability and Odontogenic Differentiation in hDPSCs

**Figure 3:**
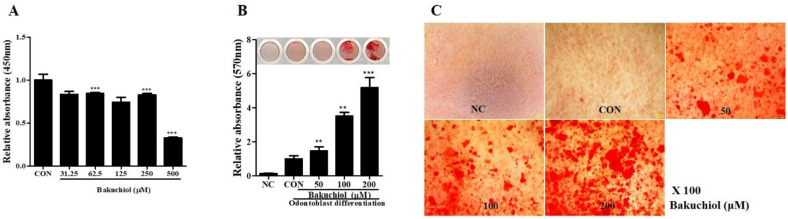
The effect of bakuchiol on proliferation and odontogenic differentiation in hDPSCs. (**A**) Cell proliferation was measured at 4 days using the CCK-8 assay. When treated with 50, 100 and 200 μM of bakuchiol, cell proliferation was comparable to the control group. *** *p* < 0.01. (**B**) Alizarin red S (ARS) staining was performed on the 10th day of odontogenic differentiation of hDPSCs treated with bakuchiol. (**C**) Scanned images of plate wells are presented above the quantitation graph of ARS staining. All data are presented as the mean ± SEM (n = 3). ** *p* < 0.02, *** *p* < 0.01. (**C**) 100× microscope images of hDPSCs treated with 50, 100 and 200 μM of bakuchiol. SEM, standard error of mean; NC, negative control; CON, positive control; hDPSCs, human dental pulp stem cells.

To evaluate the effects of bakuchiol, concentrations of 50, 100 and 200 µM were used for CCK-8 assay and odontogenic differentiation. The cell viability decreased by only 10%, except at 500 μM. The ratio was less than 20% at 250 μM. Since we wanted to choose no cell-toxicity bakuchiol concentrations, this experiment used bakuchiol concentrations of 50, 100 and 200 μM, which were reasonable. The hDPSCs were treated for 10 days with bakuchiol to induce odontogenic differentiation, and the differentiation ability was determined by ARS staining. The degree of differentiation quantified using a 10% CPC solution of each concentration is shown in a micrograph (Figure 3A–C). The results indicated that bakuchiol is a mono compound form *P. corylifolia* that can induce odontogenic differentiation.

**Figure 4:**
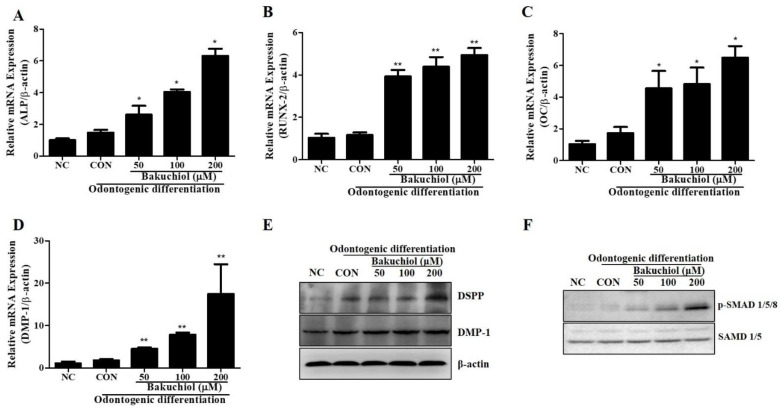
Real-time PCR analysis during odontogenic differentiation in hDPSCs. (**A**–**D**) Odontogenic differentiation marker gene expression was evaluated using real-time PCR. Expression of APL, RUNX-2, OC, and DMP-1 in hDPSCs treated with 50, 100 and 200 μM of bakuchiol. (**E**) Western blot analysis for the expression of odontogenic differentiation protein markers DMP-1 and DSPP. (**F**) Western blot analysis to determine the effect of bakuchiol on SMAD 1/5/8 phosphorylation cell signaling pathway during odontogenic differentiation. All data are presented as the mean ± SEM (n = 3). * *p* < 0.05, ** *p* < 0.02 SEM, standard error of mean; NC, negative control; CON, positive control; hDPSCs, human dental pulp stem cells; PCR, polymerase chain reaction.

The expression of differentiation markers was observed after treatment with bakuchiol during odontogenic differentiation induction. Compared to that in the control groups, the expression of ALP, RUNX-2, OC, and DMP-1 and levels of the differentiation marker proteins DMP-1 and DSPP increased in the bakuchiol-treated groups (Figure 4).

## 4. Discussion

*P. corylifolia* belongs to the legume family; the dried mature fruits of *P. corylifolia* have been widely used for their pharmacological actions in conditions such as skin diseases (leukoderma), osteoporosis [28], liver disease [33], antimicrobial activity [34], and various cancers [35,36,37,38]. In addition, it has been confirmed that bone marrow stromal cells promote osteoblast differentiation [33,39]. Based on the diversity of osteoporosis inhibition studies, we examined the induction of odontogenic differentiation by *P. corylifolia* extract in hDPSCs.

Bakuchiol, a high-content meroterpinoid of *P. corylifolia*, was reported with anti-bactericidal [40], anti-inflammatory [41], and estrogenic activities in vitro [42]. Wei et al. suggested that bakuchiol exhibited a stronger effect to enhance osteoblasts differentiation than the other components [41]. Bakuchiol has a special structure, which also has a prenyl group. The active components of *P. corylifolia* that promote bone formation, and PPARγ and hydrocarbon receptor were verified as targets of *P. corylifolia* in MC3T3-E1 cells [43]. Various studies have shown that the preservation of natural teeth is related to the extension of the human lifespan, and the placement of implants to replace the role of teeth after extraction can be considered to be revolutionary [44,45]. The odontoblast differentiation of DPSCs has been studied earlier [46]. In this study, we confirmed that *P. corylifolia* extracts promote the odontogenic differentiation of hDPSCs. The efficacy of *P. corylifolia* extracts has not yet been reported, and the discovery of a natural product that promotes odontogenic differentiation of hDPSCs is encouraging. In addition, a patent (10-2021-0088822) has been obtained based on these experimental results. As a result, it was confirmed that expression of odontogenic differentiation markers increased during differentiation in a concentration-dependent manner. Based on the results of this experiment, it is expected that it will be possible to develop a new type of pharmaceutical aid that can preserve natural teeth. In summary, *P. corylifolia* promoted odontogenic differentiation. Bakuchiol was identified as the compound in *P. corylifolia* responsible for this effect. These can have potential applications in tooth regeneration.

## 5. Conclusions

In conclusion, this research is the first suggestion that *P. corylifolia* and bakuchiol which is a mono-compound of *P. corylifolia*., are effective compound accelerate odontoblast differentiation. Up-regulated odontoblast differentiation markers genes and proteins expression and SMAD phosphorylation support our purpose.

## Figures and Tables

**Table 1:** Real-time PCR primer sequences.

Name	Sequences (5′ → 3′)	
ALP	F	GACCTCCTCGGAAGACACTC
	R	TGAAGGGCTTCTTGTCTGTG
RUNX-2	F	GGTTAATCTCCGCAGGTCACT
	R	CACTGTGCTGAAGAGGCTGTT
OC	F	GCAGCGAGGTAGTGAAGAGAC
	R	AGCAGAGCGACACCCTAGA
DMP-1	F	CAAGACAGTGCCCAAGATAC
	R	TTCCCTCATCGTCCAACT
β-actin	F	GGCACCCAGCACAATGAAG
	R	TGCGGTGGACGATGGAGG

PCR, polymerase chain reaction.

## Data Availability

Not applicable.

## Abbreviations

*P. corylifolia*	Psoralea Corylifolia Linn
hDPSCs	human dental pulp stem cells
GSK-3β	glycogen synthase kinase 3 beta
MSCs	Mesenchymal stem cells
HLA	human leukocyte antigen
DMSO	Dimethyl sulfoxide
CCK-8	Cell counting kit-8
ARS	Alizarin Red S
CPC	Cetylpyridinium chloride
PCR	polymerase chain reactin
SEM	standard error of the mean
SMAD	small mothers against decapentaplegia
